# School screening for scoliosis: can surface topography replace examination with scoliometer?

**DOI:** 10.1186/1748-7161-7-9

**Published:** 2012-04-03

**Authors:** Joanna Chowanska, Tomasz Kotwicki, Krzysztof Rosadzinski, Zbigniew Sliwinski

**Affiliations:** 1 Spine Disorders Unit, Department of Pediatric Orthopedics and Traumatology, University of Medical Sciences, Górecka 30, 60-201 Poznan, Poland; 2 Rehasport Clinic, Górecka 30, 60-201 Poznan, Poland; 3 Rehabilitation Centre, Konarskiego 5B, 59-900 Zgorzelec, Poland; 4 Jan Kochanowski University of Humanities and Sciences, Kielce, Poland

**Keywords:** Idiopathic scoliosis, Scoliosis school screening, Scoliometer, Surface topography

## Abstract

**Background:**

Clinical examination with the use of scoliometer is a basic method for scoliosis detection in school screening programs. Surface topography (ST) enables three-dimensional back assessment, however it has not been adopted for the purpose of scoliosis screening yet. The purpose of this study was to assess the usefulness of ST for scoliosis screening.

**Methods:**

996 girls aged 9 to 13 years were examined, with both scoliometer and surface topography. The Surface Trunk Rotation (STR) was introduced and defined as a parameter allowing comparison with scoliometer Angle of Trunk Rotation taken as reference.

**Results:**

Intra-observer error for STR parameter was 1.9°, inter-observer error was 0.8°. Sensitivity and specificity of ST were not satisfactory, the screening cut-off value of the surface topography parameter could not be established.

**Conclusions:**

The study did not reveal advantage of ST as a scoliosis screening method in comparison to clinical examination with the use of the scoliometer.

## Background

Idiopathic scoliosis is a three-dimensional developmental deformity of the spine. It affects about 2 - 3% of adolescents population [1-3]. Scoliosis progression occurs more frequently among girls and during puberty, which contributes to the fact that young females of 10 to 12 years old are the most susceptible to occurrence and progression of scoliosis [4].

Scoliosis screening is a broadly discussed topic [3,5-11]. Arguments against screening exist: (1) low predictive value leading to excessive number of children referred to specialists; (2) possibly increased amount of x-ray imaging in children; (3) lack of certainty about which small scoliosis (below 20° of Cobb angle) will progress and require treatment; (4) cost issue and (5) stress induced by examination [12,13]. Despite those facts, screening is the most important factor preventing from the deformity progression. It has been reported that early scoliosis detection allows early treatment with better outcome [1,5,6,9,14-17].

Scoliosis screening has not been designed to serve as a diagnostic method. Its main purpose is to select children with high probability of occurrence of idiopathic scoliosis out of total population. The most important criteria for screening test are: accuracy, reproducibility, sensitivity and specificity. The screening test should be quick, cheap, easy to perform, safe, noninvasive, acceptable and should have well-defined cut-off values [9,18-21]. The number of children positively screened (suspected of having scoliosis) should correspond to prevalence of idiopathic scoliosis in the population [7]. Children with intermediate trunk asymmetries ought to be rechecked at school within a few months as long as the asymmetry is not progressive [2,22].

**Figure 1:**
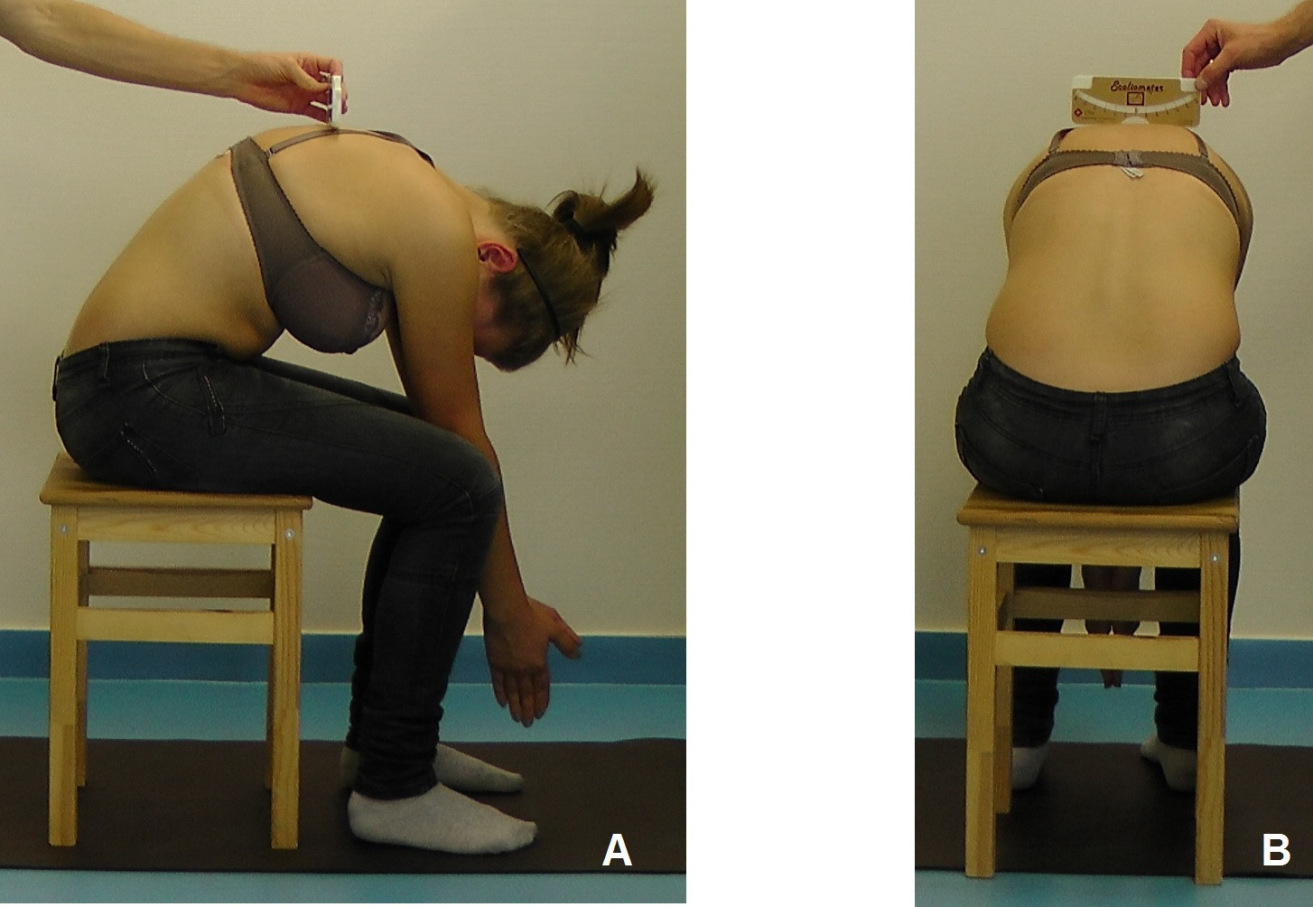
**Measurement of Angle of Trunk Rotation with Bunnell scoliometer in sitting position with forward bending: A - side view, B - posterior view**.

The gold standard for idiopathic scoliosis diagnosis is x-ray imaging, however children are not exposed to it for screening purpose, because of the radiation risk [7,23]. The basic method of school screening for scoliosis is clinical examination in forward bending position (Adams test) with the use of scoliometer which can be performed either in standing or in sitting position, Figure 1, [24,25]. The scoliometer measures the Angle of Trunk Rotation (ATR). Bunnell defined the following screening cut-off criteria [26]:

• the trunk rotation is within normal limits: ATR from 0° to 3°,

• the trunk rotation is intermediate: ATR from 4° to 6°,

• the trunk rotation is relevant and it is highly probable that the child has scoliosis: ATR ≥ 7°.

Scoliometer examination reveals good repeatability and reproducibility [27]. For the cut-off value of the ATR equal to or greater than 7° the scoliometer examination is characterized by high sensitivity (83,3%) and high specificity (86,8%) [15].

**Figure 2:**
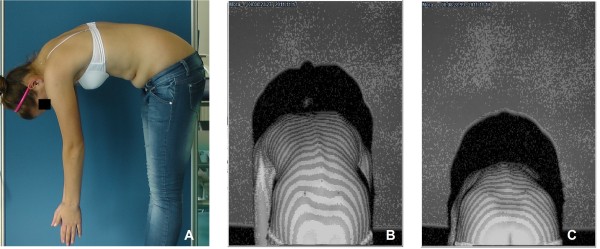
**Standing forward bending position (A) is not useful in surface topography examination - it causes the images to be captured tangentially to the back projection (B, C)**.

Surface topography (ST) is a method of trunk shape evaluation, based on external body contour assessment which can be performed with the use of several techniques. The historical moire ST was based on interference of grids projected onto subject's back [18,28-32]. Currently used methods base on computerized image capturing and digitally calculated parameters. The following techniques utilize: (1) raster stereography based on distortion of grid composed of parallel lines projected onto back [18,33-35] or (2) body scanning with light beam and its distortion analysis [18,36,37]. In our country a portable raster stereography device is available (CQ Electronic System, Wroclaw, Poland) and was used in this study. The accuracy of measurement reported by the producer equals 1 millimeter or 0.1 degree [38]. A variety of surface topography techniques, a multitude of assessed parameters together with lack of specific cut-off values, as well as limited availability of equipment seem to be main reasons why the surface topography examination is still not used for scoliosis screening. On the other hand, the accuracy of the three-dimensional assessment, the harmlessness and possibility of data storage make the surface topography examination potentially advantageous. Surface topography is usually performed in standing erect position, however it is not possible in standing position with trunk forward flexion, Figure 2.

### Purpose of the study

The purpose of this study was to assess the usefulness of surface topography method for school screening for scoliosis. Scoliometer examination was used as a reference examination.

## Methods

The study has been performed with the approval of the Poznan University of Medical Sciences Bioethics Committee, decision number 1112/08. Agreements of school principal and of parents were required prior to examination.

Examination included 996 girls between age of 9 and 13, average 11.0 ± 1.0 years. Clinical evaluation of the spine, the ATR measurement with the use of Bunnell scoliometer and the surface topography examination with the use of CQ Electronic System (Poland) device were performed on the same day by one observer (J.C.). Additionally 10 children underwent ST examination performed by 3 researchers in order to measure value of inter-observer error for Surface Trunk Rotation (STR) parameter.

Scoliometer examination required uncovering of the upper part of the body; the girls did not have to take off their bras. Scoliometer examination was performed in a sitting on a chair position with forward bending of the trunk. ATR measures were done on three levels of the spine: proximal thoracic, main thoracic and lumbar, and the maximal ATR value was retained. Number of positively screened children was determined based on the ATR ≥ 7° criterion.

**Figure 3:**
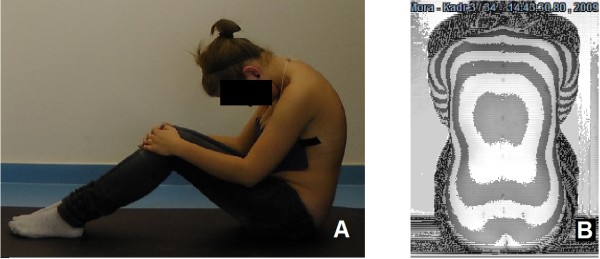
**Sitting forward bending position as used for surface topography examination: A - side view, B - posterior view as produced with surface topography**.

For surface topography examination, it was necessary to uncover the whole surface of the back and to mark anatomical landmarks: spinous processes from C7 through S1, and posterior superior iliac spines. During examination the light was turned off and the child was sitting with forward flexion of the trunk, the shoulders over the pelvis and the knees flexed at right angle, Figure 3. The projection angle was 90°, which means that the camera was placed perpendicularly to the measured surface. The 40 milliseconds images of the back were captured with a CCD camera. Recording of a sequence of images took from 5 to 15 seconds then one image, the most characteristic to the child was chosen for further analysis.

**Figure 4:**
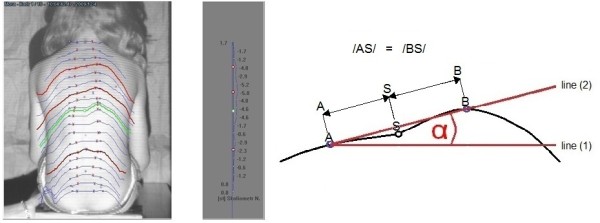
**Surface Trunk Rotation (STR) parameter setting**. Left - surface trunk rotation on nineteen spine levels, from which the maximal value is automatically selected and named STR. Right - scheme showing the surface trunk rotation angle determination: the line (1) is situated within the frontal plane, the line (2) connects two points: A and B situated at the surface at equal distance from the corresponding spinous process;/AB/is equal to the distance between two PSIS,/AS/is equal to half a distance between two PSIS,/AS/is equal to/BS/.

On each spine level from C7 through S1 the angle of surface rotation (α angle) is contained between two adjacent lines: (1) a line situated within the frontal plane and (2) a line which connects two points lying on the back surface, situated symmetrically on the left and on the right side of the corresponding spinous process. The distance between the two points (point A and point B) was defined to be equal to the distance between the two posterior superior iliac spines of the patient (PSIS). The distance between each point and spinous process (S) is equal to half a distance between two PSIS, Figure 4. The maximal value of the trunk rotation was named the Surface Trunk Rotation (STR) and was automatically picked up with the dedicated software as the highest rotation value of 19 spine levels from C7 to S1.

The repeatability of ST examination was assessed based on the value of intra-observer error and inter-observer error [39]. The intra-observer error for the STR parameter was assessed based on examination performed twice by the same researcher in the group of 50 girls (100 examinations in total). There was a break between examinations to perform several bends, jumps, arms swings and sit-ups. The value of inter-observer error for STR was assessed in the group of ten girls examined by three researchers. Each girl was examined once by each researcher (30 examinations in total) and a break was taken between examinations.

Surface topography measurement results were compared to scoliometer measurement results treating scoliometer measurement as a reference. Assessment of the repeatability, sensitivity, specificity, positive predictive value and negative predictive value of surface topography measurement was performed, assuming the value of ATR ≥ 7° as the reference value. The calculations were performed four times, for the Surface Trunk Rotation of 7, 6, 5 and 4 degrees, respectively. Time required for performing surface topography examination and scoliometer examination was assessed.

## Results

The number of girls positively screened with the use of scoliometer (ATR value greater than or equal to 7°) was 45, the percentage was 4.5%.

Out of 996 subjects, 21 results of surface topography (2.1%) had to be excluded from analysis because of the surface topography image artifacts. No patient had to be excluded from the scoliometer examination analysis. For the STR parameter the value of intra-observer error was 1.9° and the value of inter-observer error was 0.8°.

The number of children presenting true positive, true negative, false positive, false negative results of surface topography examination in relation to the Bunnell scoliometer examination is presented in Table 1.

**Table 1:** Results of surface topography measurement in relation to the Bunnell scoliometer measurement

ATR scoliometer	STR surface topography	True positive	False positive	False negative	True negative
≥ 7°	≥ 7°	12	13	19	931

≥ 7°	≥ 6°	15	42	16	902

≥ 7°	≥ 5°	20	113	11	831

≥ 7°	≥ 4°	24	273	7	671

ATR - angle of trunk rotation, STR - surface trunk rotation.

The number of children presented with the true positive, true negative, false positive, false negative results

The sensitivity, specificity, positive predictive values (PPV) and negative predictive values (NPV) of the STR are presented in Table 2.

**Table 2:** Sensitivity, specificity, positive and negative predictive value of surface trunk rotation related to ATR ≥7°

ATR scoliometer	STR surface topography	SENSITIVITY [%]	SPECIFICITY [%]	PPV [%]	NPV [%]
≥ 7°	≥ 7°	38.7	98.6	48.0	98.0

≥ 7°	≥ 6°	48.4	95.6	26.3	98.3

≥ 7°	≥ 5°	64.5	88.0	15.0	98.7

≥ 7°	≥ 4°	77.4	71.1	8.1	99.0

ATR- angle of trunk rotation, STR -surface trunk rotation, PPV -positive predictive value, NPV -negative predictive value.

The ATR measurement lasted around 2 minutes (from 1 to 3 minutes) per child. Surface topography evaluation with image assessment lasted about 10 minutes (from 7 to 15 minutes) per child.

## Discussion

In this study, the percentage of girls positively screened with the use of scoliometer (ATR value greater than or equal to 7°) corresponded with the literature data: Bunnell: 2-3% [2], Fong: 0.1-7.45% [3], Yawn: 4.1% [22], Korovessis: 4.37% [40].

As it is recommended to perform clinical examination in forward bending position for obtaining better visualization of spine alignment and trunk rotation, we choose the forward bending position during ST examination for the same reason. Traditionally, scoliometer examination is performed in standing forward bending position while surface topography in standing erect posture. In this study, both examinations were performed in sitting position with trunk flexion. Consequently, the trunk rotation parameters of both examinations (scoliometer Angle of Trunk Rotation and surface parameter Surface Trunk Rotation) could be compared. Other advantages of the sitting position are the posture stability and no impact of lower limbs discrepancy on the pelvis level.

Surface topography was reported to measure precisely the trunk asymmetry [34,41-43].

This study revealed the following disadvantages of surface topography method in scoliosis screening:

a) difficulty to definite the cut-off values for the surface topography parameter (STR),

b) unsatisfactory sensitivity and specificity of the surface topography examination,

c) the ST examination was more complex than scoliometer examination and it required longer training,

d) the children had to uncover completely their back for the ST examination,

e) the ST examination took five times longer than evaluation with the use of scoliometer - because it requires longer preparation associated with full uncovering of subject's back, marking of relevant points on it, image selection and evaluation,

f) necessity of the ST equipment delivery, the room adaptation and access to a computer,

g) estimated cost of the ST device used in this study was equal to the cost of 280 scoliometers.

**Figure 5:**
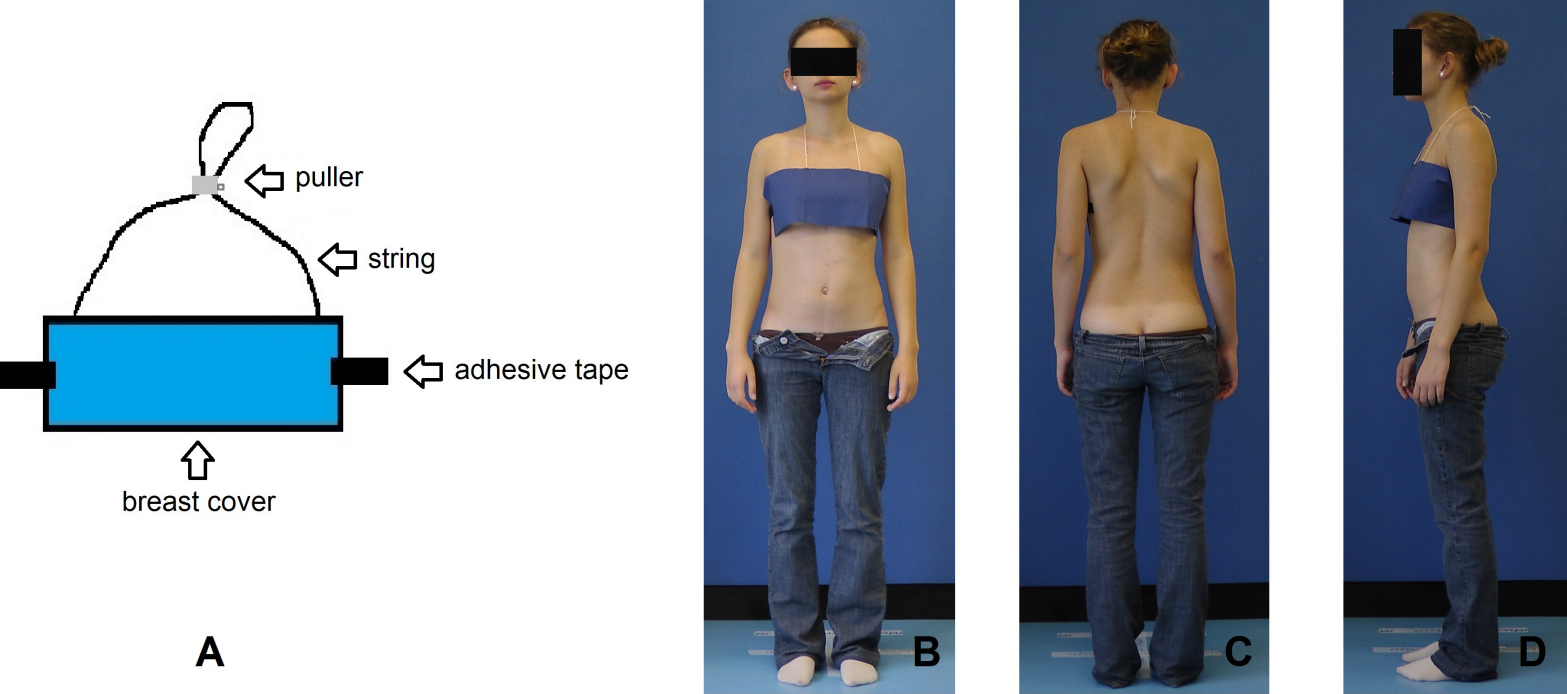
**Breast cover: A - scheme, B - anterior view, C - posterior view, D - lateral view**.

During surface topography examination, the need to uncover the whole surface of the back turned out to be problematic, especially for adolescent girls in school environment. To overcome this problem we used a screen to separate the examination area as well as a specially constructed disposable breast cover for girls, Figure 5.

Surface topography evaluation allows examination of patients in both upright and forward bending positions. The sitting position with trunk flexion allows to evaluate trunk rotation (Figure 3), so the results can be compared with scoliometer examination results. Other advantages of the sitting position are the posture stability and no impact of lower limbs discrepancy on the pelvis level.

The intra-observer error for STR parameter was 1.9° which was higher than that of the inter-observer error (0.8°). The possible reason can be related to methodological differences in calculating both errors. There were more patients taken into account for the purpose of calculation of the intra-observer error (50 children examined twice by one observer, which gives 100 values) than for the calculation of the inter-observer error (10 children examined once by 3 observers which gives 30 values).

Based on the trunk rotation assessment results we can state that CQ surface topography evaluation has a good repeatability and reproducibility. However, it was not possible to choose a reasonable cut-off value of the surface topography parameter. For the value of STR ≥ 5° the sensitivity was 64.5% and the specificity was 88%. For the value of STR ≥ 4° the sensitivity was 77.4% and the specificity was 71.1%. No STR value provided simultaneously a satisfactory sensitivity and a satisfactory specificity.

One of the limitations of the study is that the children did not undergo radiographic examination. Although scoliometer has been widely used for screening purpose, it still has its own limitations. It may not be fully reliable as a standard for comparison of surface topography because the ultimate assessment of scoliosis currently depends on radiography.

Based on the available data [8-10,12,16,17], the estimated cost of school screening for scoliosis ranges from less than USD 1.00 to more than USD 30.00 per child screened. The lower estimates considered calculations for conducting screening program *per se*, borne by the screening centers or schools. The higher estimates include the induced medical care costs: health care visits and tests resulted from referral recommendations. Our own observations suggest that the use of scoliometer can decrease the cost of school screening for scoliosis and the use of surface topography increase it.

## Conclusions

The study did not reveal advantage of the surface topography as a screening method in detection of idiopathic scoliosis in comparison to clinical examination with the use of the scoliometer.

## Abbreviations

ATR: Angle of trunk rotation; ST: Surface topography; STR: Surface trunk rotation; C7: Seventh cervical spinous process; S1: First sacral spinous process; PPV: Positive predictive value; NPV: Negative predictive value; USD: United States dollar; PSIS: Posterior Superior Iliac Spine.

## Competing interests

The authors declare that they have no competing interests.

## Authors' contributions

JC - performed children examination, data analysis and drafted the manuscript. TK - conceived of the study, participated in its design, data interpretation and drafting of the manuscript. KR - helped in research coordination, assisted in children examination and data analysis. ZS - participated in data interpretation and manuscript corrections. All authors read and approved the final manuscript.

## References

[B1] Asher MA Burton DC Adolescent idiopathic scoliosis: natural history and long term treatment effects Scoliosis 2006 1 2 16759428 10.1186/1748-7161-1-2 PMC1475645

[B2] Bunnell W Selective screening for scoliosis Clin Orthop Relat Res 2005 434 40 45 15864030 10.1097/01.blo.0000163242.92733.66

[B3] Fong DY Lee CF Cheung KM Cheng JC Ng BK Lam TP Mak KH Yip PS Luk KD A meta-analysis of the clinical effectiveness of school scoliosis screening Spine 2010 35 10 1061 1071 20393399 10.1097/BRS.0b013e3181bcc835

[B4] Wong HK Hui JH Rajan U Chia HP Idiopathic scoliosis in Singapore schoolchildren: a prevalence study 15 years into the screening program Spine 2005 30 10 1188 1196 15897834 10.1097/01.brs.0000162280.95076.bb

[B5] Fazal M Edgar M Detection of adolescent idiopathic scoliosis Acta Orthop Belg 2006 72 184 186 16768263

[B6] Grivas TB Wade MH Negrini S O'Brien JP Maruyama T Hawes MC Rigo M Weiss HR Kotwicki T Vasiliadis ES Sulam LN Neuhous T Sosort consensus paper: school screening for scoliosis: Where are we today? Scoliosis 2007 2 17 18039374 10.1186/1748-7161-2-17 PMC2228277

[B7] Richards SB Vitale MG Screening for Idiopathic Scoliosis in Adolescents: An Information Statement J Bone Joint Surg 2008 90 195 198 18171974 10.2106/JBJS.G.01276

[B8] Yawn BP Yawn RA The estimated cost of school scoliosis screening Spine 2000 25 2387 2391 10984793 10.1097/00007632-200009150-00019

[B9] Grivas TB Vasiliadis ES Maziotou C Savvidou OD The direct cost of "Thriasio" school screening program Scoliosis 2007 2 7 17501989 10.1186/1748-7161-2-7 PMC1876446

[B10] Lee CF Fong DY Cheung KM Cheng JC Ng BK Lam TP Mak KH Yip PS Luk KD Costs of school scoliosis screening: a large, population-based study Spine 2010 35 26 2266 2272 20531070 10.1097/BRS.0b013e3181cbcc10

[B11] Cilli K Tezeren G Taş T Bulut O Oztürk H Oztemur Z Unsaldi T School screening for scoliosis in Sivas, Turkey Acta Orthop Traumatol Turc 2009 43 5 426 430 19881324 10.3944/AOTT.2009.426

[B12] Morais T Bernier M Turcotte F Age- and sexspecific prevalence of scoliosis and the value of school screening programs Am J Public Health 1985 75 1377 1380 4061707 10.2105/ajph.75.12.1377 PMC1646463

[B13] U.S. Preventive Services Task Force (USPSTF) Recommendation statement: screening for idiopathic scoliosis in adolescents 2004 http://www.uspreventiveservicestaskforce.org/3rduspstf/scoliosis/scoliors.pdf

[B14] Torell G Nordwall A Nachemson A The changing pattern of scoliosis treatment due to effective screening J Bone Joint Surg Am 1981 63 337 341 7204428

[B15] Ashworth MA Hancock JA Ashworth L Tessier KA Scoliosis screening. An approach to cost/benefit analysis Spine 1988 13 1187 1188 3144755 10.1097/00007632-198810000-00024

[B16] Thilagaratnam S School-based screening for scoliosis: is it cost-effective? Singapore Med J 2007 48 11 1012 1017 17975691

[B17] Lonstein JE Bjorklund S Wanninger MH Nelson RP Voluntary school screening for scoliosis in Minnesota J Bone Joint Surg Am 1982 64 4 481 488 6802853

[B18] McCarthy RE Evaluation of the patient with deformity Weinstein S, red. The Pediatric Spine 1994 New York: Raven 185 224

[B19] Morrissy RT School screening for scoliosis: a statement of the problem Spine 1988 13 10 1195 1197 3206277 10.1097/00007632-198810000-00028

[B20] Williams J Criteria for screening: are the effects predictable? Spine 1988 13 10 1178 1186 3144754 10.1097/00007632-198810000-00023

[B21] Wilson JMG Jungner F Principles and practice of screening for disease 1968 Public Health Papers No. 34 Geneva: World Health Organization 1 39

[B22] Yawn BP Yawn RA Hodge D Kurland M Shaughnessy WJ Ilstrup D Jacobsen SJ A population-based study of school scoliosis screening JAMA 1999 282 1427 1432 10535432 10.1001/jama.282.15.1427

[B23] Dutkowsky JP Shearer D Schepps B Orton C Scola F Radiation exposure to patients receiving routine scoliosis radiography measured at depth in an anthropomorphic phantom J Pediatr Orthop 1990 10 4 532 534 2358494

[B24] Grivas TB Vasiliadis ES Mihas C Triantafyllopoulos G Kaspiris A Trunk asymmetry in juveniles Scoliosis 2008 3 13 18811937 10.1186/1748-7161-3-13 PMC2590591

[B25] Kotwicki T Chowanska J Kinel E Lorkowska M Stryła W Szulc A Sitting forward bending position versus standing position for studying the back shape in scoliotic children Scoliosis 2007 2 1 34

[B26] Bunnell W Outcome of Spinal Screening Spine 1993 18 12 1572 1580 8235833 10.1097/00007632-199309000-00001

[B27] Amendt LE Ause-Ellias KL Eybers JL Wadsworth CT Nielsen DH Weinstein SL Validity and reliability testing of the scoliometer Phys Ther 1990 70 2 108 117 2296610 10.1093/ptj/70.2.108

[B28] Willner S Development of trunk asymmetries and structural scoliosis in prepubertal school children in Malmo: follow-up study of children 10-14 years of age J Pediatr Orthop 1984 4 Raven Press, New York 452 455 10.1097/01241398-198408000-00012 6470115

[B29] Daruwalla J Balasubramaniam P Moire topography in scoliosis J Bone Joint Surg 1985 67B 211 213 10.1302/0301-620X.67B2.3980527 3980527

[B30] Ruggerone M Austin J Moire topography in scoliosis: correlations with vertebral lateral curvature as determined by radiography Phys Ther 1986 66 7 1072 1107 3725892 10.1093/ptj/66.7.1072

[B31] Adair IV Van Wijk MC Armstrong GW Moiré topography in scoliosis screening Clin Orthop Relat Res 1977 129 165 171 608270 10.1097/00003086-197711000-00019

[B32] Adler N Csongradi J Bleck E School Screening for Scoliosis-One Experience in California Using Clinical Examination and Moiré Photography West J Med 1984 141 5 631 633 6516333 PMC1011169

[B33] Drerup B Hierholzer E Ellger B Sevastik JA, Diab KM Shape analysis of the lateral and frontal projection of spine curves assessed from rasterstereographs Research Into Spinal Deformities 1997 1 Amsterdam, The Netherlands: lOS Press 271 275

[B34] Gibeault JP Fast and radiation-free technology for spine and pelvis analysis Biometrix Medica 2008 http://www.biometrixmedica.com/en/resources/white.papers.html

[B35] Zubairi J Applications of computer-aided rasterstereography in spinal deformity detection Image Vis Comput 2002 20 319 324

[B36] Upadhyay SS Burwell RG Webb JK Hump changes on forward flexion of the lumbar spine in patients with idiopathic scoliosis Spine 1988 13 2 146 151 3406833 10.1097/00007632-198802000-00003

[B37] Turner-Smith AR Harris JD Houghton GR Jefferson RJ A method for analysis of back shape in scoliosis J Biomech 1988 21 6 497 509 3209594 10.1016/0021-9290(88)90242-4

[B38] CQ Electronic System http://www.cq.com.pl

[B39] Hopkins W Measures of reliability in sports medicine and science Sports Med 2000 30 1 1 15 10907753 10.2165/00007256-200030010-00001

[B40] Korovessis P Stamatakis M Prediction of scoliotic Cobb angle with the use of the scoliometer Spine 1996 21 1661 1666 8839469 10.1097/00007632-199607150-00010

[B41] Oxborrow N Assessing the child with scoliosis: the role of surface topography Arch Dis Child 2000 83 453 455 11040160 10.1136/adc.83.5.453 PMC1718548

[B42] Patias P Grivas TB Kaspiris A Aggouris C Drakoutos E A review of the trunk surface metrics used as Scoliosis and other deformities evaluation indices Scoliosis 2010 5 12 20584340 10.1186/1748-7161-5-12 PMC2906414

[B43] Pazos V Cheriet F Song L Labelle H Dansereau J Accuracy assessment of human trunk surface 3D reconstructions from an optical digitising system Med Biol Eng Comput 2005 43 11 15 15742714 10.1007/BF02345117

